# Surgical excision of cerebral glioma and multimodal treatment including Cerebrolysin: a case report

**DOI:** 10.25122/jml-2025-0142

**Published:** 2025-11

**Authors:** Jose Daniel Salvador Ruiz González, Andrea Tapia

**Affiliations:** 1 Department of Neurosurgery, Fundación Clínica Médica Sur, Ciudad de Mexico, Mexico; 2 Instituto Mexicano del Seguro Social , Hospital General Zona 1, Cuernavaca Morelos, Mexico

**Keywords:** cerebral glioma, Cerebrolysin, brain protection, multimodal neurooncological treatment

## Abstract

Multimodal treatment of patients with a brain tumor primarily involves microsurgical excision, ideally radical, or at least subtotal resection. Tumors in deep or inaccessible locations may require biopsy followed by adjuvant therapy with chemotherapy, radiotherapy, or radiosurgery. Beyond controlling tumor growth, preserving neurological function and promoting brain plasticity are essential goals. Persistent inflammation and intracranial hypertension can trigger secondary injury through edema, excitotoxicity, and ischemia, potentially resulting in irreversible neuronal damage. Multimodal strategies, including neuroprotective measures such as Cerebrolysin administration, may help prevent or mitigate these secondary processes, supporting recovery and functional outcomes. Clinically, patients may present with headache, nausea, vomiting, seizures, or subtle cognitive and motor deficits, progressing in severe cases to deterioration of consciousness. Incorporating cerebroprotective interventions in perioperative management represents a promising approach to enhance recovery, functional independence, and quality of life in neurosurgical oncology patients.

## Introduction

Gliomas represent one of the most challenging tumors in neurosurgery due to their infiltrative growth pattern, high recurrence rate, and the difficulty of achieving complete surgical excision without causing significant neurological deficits. Despite advances in microsurgical techniques, intraoperative navigation, and perioperative multimodal management, the prognosis of patients with high-grade gliomas remains poor, with survival and quality of life significantly affected by the balance between aggressive tumor resection and preservation of neurological function [1].

Tumor resection, whether subtotal or radical, is often accompanied by peri-tumoral edema, ischemia, and secondary inflammatory cascades that compromise the viability of adjacent tissue. This so-called 'penumbra zone' represents an area of vulnerable but potentially salvageable brain parenchyma [2,3]. Protecting this zone and enhancing functional recovery are therefore crucial objectives in glioma surgery.

In recent years, the concept of cerebroprotection has gained increasing relevance, not only in acute stroke and traumatic brain injury but also in the context of planned neurosurgical interventions [4-9]. While cranial surgery for tumor resection is a controlled procedure, it invariably produces mechanical and biochemical stress comparable to brain trauma. Surgical manipulation, vascular compromise, ischemia–reperfusion injury, and postoperative edema all contribute to neuronal dysfunction and the risk of long-term disability [10,11]. Thus, the application of cerebroprotective agents during and after glioma surgery offers a rational therapeutic strategy to minimize iatrogenic damage, facilitate neuroplasticity, and improve clinical outcomes [5,7,9,12].

Cerebrolysin, a neuropeptide preparation with documented neuroprotective and neurorestorative properties, has shown promise in conditions such as traumatic brain injury, stroke, and neurodegenerative diseases [4-9,13,14]. Its mechanisms include the modulation of neurotrophic signaling, anti-apoptotic effects, reduction of excitotoxicity, and promotion of synaptic remodeling and neurogenesis [1]. Translating these benefits into the multimodal management of glioma patients represents a novel but logical extension of its therapeutic use.

We present a case in which multimodal treatment, including Cerebrolysin, was employed as an adjuvant treatment following surgical resection of an anaplastic astrocytoma. This report highlights the potential role of cerebroprotection in planned neurosurgical oncology, aiming to optimize patient recovery and quality of life beyond the oncological control of the disease.

## Patient information

A 50-year-old woman with no significant medical history presented with severe, disabling headaches unresponsive to analgesics. Neurological assessment and MRI revealed a temporal lobe intracranial tumor. In October 2019, a biopsy confirmed an anaplastic astrocytoma, Grade III. The patient subsequently underwent subtotal tumor resection, followed by adjuvant chemotherapy with Temozolomide (200 mg/m^2^ daily for 42 days) and holocranial radiotherapy (3 Gy/day for 10 days) at another hospital.

The patient came for a second opinion, with imaging findings consistent with a left temporal lesion suggestive of residual or recurrent glioma, resistant to previous radio- and chemotherapy.

**Figure 1:**
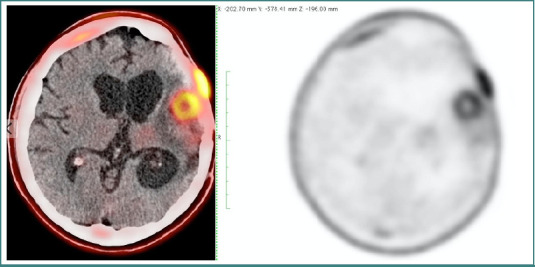
Pet CT scan FDG. Hypermetabolic temporal lesion compatible with residual or recurrent lesion.

Follow-up imaging, PET-CT, documented a left fronto-temporal residual lesion with extensive cerebral hypoperfusion and ischemia secondary to the tumor, as well as hypermetabolic areas corresponding to residual-recurrent tumor (Figure 1). Neurological evaluation revealed intact cranial nerves and cerebellar function, but right hemicorporal paresis (3/5 on the Daniels scale) with preserved muscle stretch reflexes.

**Figure 2:**
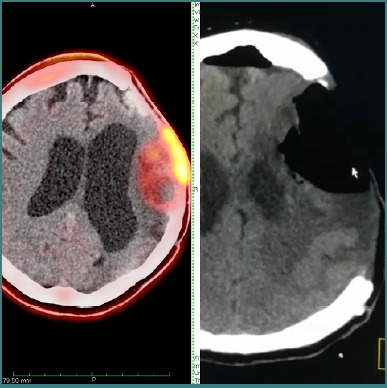
Pre- and postoperative cranial tomography

**Figure 3:**
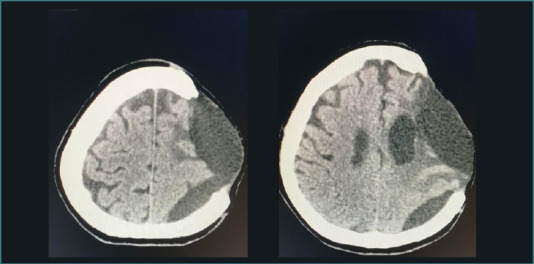
Simple cranial tomography after decompressive craniotomy

The second surgical procedure was performed without intraoperative complications (Figure 2). Two weeks after the second glioma resection, the patient developed cerebral edema that was unresponsive to conservative management, including conventional doses of dexamethasone. Consequently, a decompressive craniectomy with duroplasty was performed (Figure 3). Following this procedure, antibiotic and analgesic therapy were included in the postoperative treatment plan.

**Figure 4:**
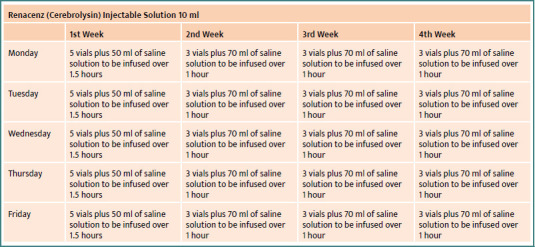
Cerebrolysin schedule

After a multidisciplinary discussion involving the oncology, internal medicine, and neurosurgery teams, it was decided to initiate Cerebrolysin therapy immediately after surgery. Before administering the treatment, informed consent was obtained from the patient’s family. The patient received Cerebrolysin according to the treatment schedule shown in Figure 4.

At home, 3 days after surgery, the patient began a physical rehabilitation program as outlined below.

She attended sessions three times per week in the afternoons. During the initial assessment, she presented with right-sided hemiplegia—the right upper limb was in the flaccid stage, while the right lower limb exhibited hypotonia with no activity in the anterior tibial muscle. Her emotional state was characterized by reluctance and poor cooperation with treatment.

Rehabilitation was initiated using proprioceptive neuromuscular facilitation (PNF) techniques, functional exercises, Russian current electrostimulation, stretching of muscle chains, and both passive and active joint mobility exercises. The main objectives were to restore trunk control, mobility, and strength in the right lower limb to improve gait and facilitate transfers (e.g., from bed to wheelchair and from wheelchair to dining area), while also addressing recovery of the right upper limb.

Over the following 4 weeks, the patient showed positive progress. In the right lower limb, active mobility was achieved with limited hip range of motion, knee hyperextension, and a plantar-pointed foot position, but with improved mobility in all five toes, indicating the onset of an extensor synergy. The right upper limb also demonstrated improvement, with activation of the deltoid and biceps muscles, leading to a flexor synergy pattern that enabled active elbow flexion and finger flexion with thumb opposition.

A few months later, with continued rehabilitation, the patient was able to walk with the assistance of a four-point cane and an ankle-foot orthosis (AFO), which helped prevent tripping. Due to knee hyperextension, a knee brace with adjustable side bars was used, enabling the patient to ambulate approximately 20 meters.

In the right upper limb, improvements were also noted: shoulder flexion reached 30°, shoulder extension 15°, shoulder abduction 20°, and elbow flexion 90°. Recovery of finger extension and hand opening progressed more slowly compared to the lower limb. However, rehabilitation was temporarily suspended due to the COVID-19 pandemic.

**Figure 5:**
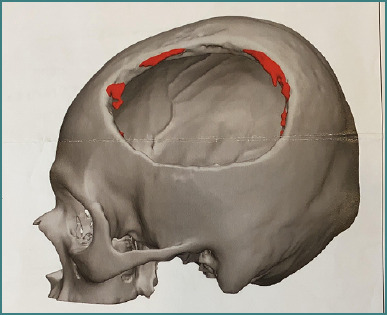
Cranioplasty with Peek's mesh

Finally, a cranioplasty using a PEEK mesh was performed, with a favorable outcome during the late postoperative period (Figure 5).

**Figure 6:**
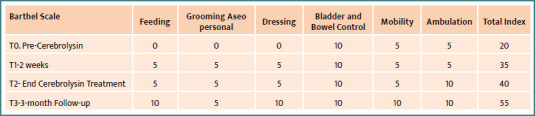
Barthel Index before and after Cerebrolysin multimodal adjuvant treatment

Clinical observations revealed a significant improvement in motor function, particularly in manual force, as assessed by the Daniels scale (improved from 3 to 4.5 points), and in independent standing and walking, as reflected in Barthel Index scores before and after Cerebrolysin treatment (Figure 6).

The patient's neurological state improved considerably, as evidenced by the clinical assessment, leading to a notable improvement in her quality of life and that of her caregivers.

**Figure 7:**
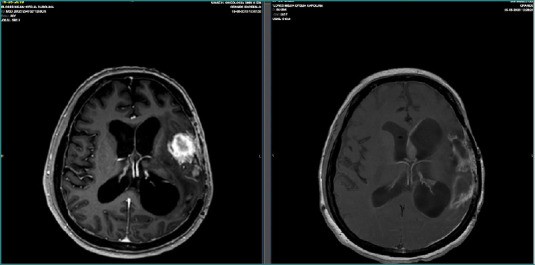
Axial preoperative and postoperative MRI scans obtained 8 months after multimodal treatment.

**Figure 8:**
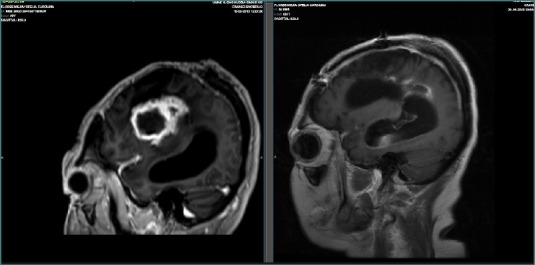
Sagittal MRI images before and after multimodal treatment

**Figure 9:**
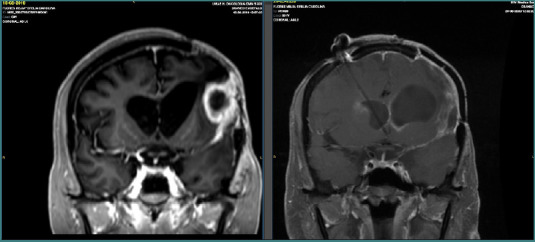
Coronal MRI images before and after multimodal treatment

Preoperative and postoperative MRI follow-up was conducted, with an 8-month interval between the first and second postoperative scans following multimodal treatment. The follow-up MRI revealed no evidence of residual tumor or recurrence, showing only postsurgical changes (Figures 7–9).

The patient demonstrated favorable clinical progress compared with her preoperative condition, achieving independence in daily activities and self-feeding. However, during subsequent clinical and radiological follow-up, the patient contracted COVID-19 and unfortunately passed away due to complications related to the infection.

## Discussion

The conventional management of cerebral edema and ischemia following tumor resection relies on corticosteroids, osmotic agents, and in severe cases, decompressive craniectomy [8]. These strategies, while effective in reducing acute intracranial hypertension, do not address the long-term mechanisms of neuronal injury, including excitotoxicity, oxidative stress, and apoptosis [15]. Moreover, they do not actively promote brain repair or neuroplasticity. In this regard, cerebroprotective and neurorestorative therapies offer a valuable adjunct to standard care [5,7,9,12].

Cerebrolysin has been extensively studied in the setting of traumatic brain injury and ischemic stroke, where its administration has been associated with improved functional recovery, reduced lesion expansion, and enhanced cognitive outcomes [4-9]. The underlying rationale is that acute brain injury—whether traumatic, vascular, or surgical—triggers a similar cascade of secondary damage, involving glutamate excitotoxicity, mitochondrial dysfunction, free radical production, and activation of pro-inflammatory pathways [16,17]. Glioma surgery, though elective, shares many of these pathophysiological features due to tumor-induced mass effect, peri-tumoral ischemia, and surgical manipulation [1].

In the present case, the administration of Cerebrolysin as a multimodal strategy following tumor resection and decompressive surgery could be related to functional improvement in motor strength and independence, despite the unfavorable baseline prognosis of a high-grade glioma. While this outcome cannot be attributed solely to pharmacological intervention, it suggests that the cerebroprotective therapy may have contributed to the rescue of peri-lesional tissue and to the enhancement of neuroplasticity during recovery [5,7,9,12].

The relevance of cerebroprotection and multimodal treatment in planned neurosurgery lies not only in minimizing immediate postoperative deficits but also in improving long-term quality of life.

With advances in oncological therapies extending survival, functional preservation has become an equally important goal of care. Patients surviving with profound neurological deficits face significant limitations, caregiver burden, and reduced quality of life. Therefore, multimodal treatment and the incorporation of neuroprotective strategies in perioperative protocols may help shift outcomes from mere survival to meaningful recovery [1].

Importantly, the field lacks randomized controlled trials evaluating the role of Cerebrolysin or similar agents in brain tumor surgery. Most evidence is derived from stroke and traumatic brain injury literature, and extrapolation to glioma surgery remains hypothetical [4-7]. Nonetheless, the overlapping mechanisms of injury provide a strong rationale for such investigations. Furthermore, as surgery-induced brain trauma is predictable and temporally controlled, it represents an ideal setting to test prophylactic and perioperative cerebroprotective interventions [1].

Future research should focus on establishing standardized multimodal treatment protocols and exploring the role of neuroprotective and neurorestorative agents, such as Cerebrolysin, within integrated strategies that include surgery, rehabilitation, and neurorehabilitation. Until then, individual cases such as the one presented here may help generate hypotheses and encourage the design of controlled clinical studies.

## Conclusion

The multimodal treatment, including surgical resection, rehabilitation, and neurorestorative therapy, demonstrates that, beyond tumor resection and standard oncological therapies, the use of cerebroprotective agents may support functional recovery in patients undergoing complex neurosurgical procedures.

While the role of cerebroprotection in planned neurosurgery is not yet established, our early experience suggests that it could represent an important adjunct to multimodal care.

Future controlled studies in brain tumor patients are warranted to determine the efficacy, safety, and optimal integration of Cerebrolysin into perioperative treatment protocols.
